# A real-time gesture recognition system using near-infrared imagery

**DOI:** 10.1371/journal.pone.0223320

**Published:** 2019-10-03

**Authors:** Tomás Mantecón, Carlos R. del-Blanco, Fernando Jaureguizar, Narciso García

**Affiliations:** Grupo de Tratamiento de Imágenes, Information Processing and Telecommunications Center and ETSI Telecomunicación, Universidad Politécnica de Madrid, Madrid, Spain; National University of Sciences and Technology, PAKISTAN

## Abstract

Visual hand gesture recognition systems are promising technologies for Human Computer Interaction, as they allow a more immersive and intuitive interaction. Most of these systems are based on the analysis of skeleton information, which is in turn inferred from color, depth, or near-infrared imagery. However, the robust extraction of skeleton information from images is only possible for a subset of hand poses, which restricts the range of gestures that can be recognized. In this paper, a real-time hand gesture recognition system based on a near-infrared device is presented, which directly analyzes the infrared imagery to infer static and dynamic gestures, without using skeleton information. Thus, a much wider range of hand gestures can be recognized in comparison with skeleton-based approaches. To validate the proposed system, a new dataset of near-infrared imagery has been created, from which good results that outperform other state-of-the-art strategies have been obtained.

## Introduction

In recent years, the number of works proposing new experiences for Human Machine Interaction (HMI) have considerably increased, especially those based on visual gesture recognition. In [1], a rehabilitation application to improve upper limb activity and mobility is presented. It uses a near-infrared visual device that also provides hand-skeleton information. Similarly, hand-skeleton information has been used to control the radio inside a car in the field of driver assistance [2]. Another example is the virtual reality sickness simulator presented in [3], which makes use of hand-skeleton information provided by the Leap Motion device to interact with the virtual environment. In [4], a structured light device providing depth-base information is used to operate a robot by hand gestures for rescue operations. A solution to manage an Unmanned Aerial Vehicle (UAV) is introduced in [5], which uses depth information to recognize hand gestures. A hand gesture recognition system based on depth imagery is used in [6] to interact with a computer.

Several visual devices have been used for the task of hand gesture recognition, providing color, depth, and infrared imagery. Some of them also provide higher semantic information, such a hand or body skeleton. This is a kind of mathematical model that represents a hand / body (in a very abstract form) by a set of vertices and edges (a graph), which encodes the position of the bones and the joints in 3D. No appearance information is considered in this case. In this regard, one of the most successful devices is the Leap Motion, which was the first one to introduce a detailed skeleton of both hands, unlike other popular visual devices that only provided high-level skeleton information of the whole body (Kinect version 1 and 2, or Asus Xtion). More specifically, Leap Motion provides a 23-node hand-skeleton model in comparison with the 3-node hand model of Kinect version 2.

The Leap Motion device also provides near-infrared stereo images with a resolution of 640 × 240 pixels and a variable frame rate between 30 and 200 fps. However, most of the works that use the Leap Motion only use the hand-skeleton information, which is obtained from the near-infrared images by a proprietary software. There are two main reasons for this fact. The first one is that processing image-based information is much complex than processing skeleton information. Therefore, the use of skeleton information allows for faster and easier deployment of HMI applications. The other reason is the strong geometrical distortion caused by the wide field of view of the optics of the embedded cameras. This fact radically changes the appearance of the object inside the image. The root of the problem is twofold. First, from a human perspective, the acquired hand images have a different geometry / appearance from the one perceived by a human, posing problems with the existing analysis algorithms that assume a human-perception-based representation. Second, from a machine-learning perspective, the hand appearance strongly changes according to its location inside the image (which does not occur with conventional cameras), leading to extra-complexity in the feature-based characterization of the different hand gestures. This can be considered as a new degree of freedom that makes more challenging the recognition / classification due to the higher intra-class variance, which ultimately results in a loss of performance.

Nonetheless, the use of the near-infrared images of the Leap Motion has potentially four advantages over image devices with human-like field of views, which appropriately exploited can tilt the scale in favour of Leap Motion. The first one is its significantly wider field of view in comparison with other devices, allowing a high degree of movement of the hand in close interaction situations. Despite the geometric distortions introduced, this wide field of view is important for allowing a natural and friendly interaction, which is not able with cameras with a standard field of view due to the reduced area of interaction that makes very difficult to perform the gestures inside the sensed area. The second advantage is its superior frame rate, up to 200 fps, with regard to other devices. Thus, the Leap Motion not only has more information available to perform the gesture recognition, but also it is able to recognize high-speed gestures. The third one is its high Signal-to-Noise Ratio (SNR) and low clutter conditions attributed to its hardware design. The Leap Motion is an active device that emits near-infrared light through three Light Emitting Diodes (LEDs), illuminating the very close objects (in this case the hands), whereas the undesired background regions are totally dark or very dimmed. Moreover, this active illumination renders images without blurring, avoiding the degradation in the hand gesture recognition performance. The last advantage is that a wider range of gestures can be potentially recognized by directly processing the provided near-infrared imagery. The reason is that the robust extraction of the skeleton information from near-infrared images is only possible for a subset of hand poses (mainly due to inter-finger occlusions), and therefore it restricts the range of gestures that can be recognized using the skeleton. On the other hand, there are works that have proven that near-infrared imagery is especially useful to detect human skin, developing solutions for human [7] and vehicle occupants’ detection [8].

In this paper, a novel real-time hand gesture recognition system that only uses the near-infrared images of the Leap Motion device is presented, which fully exploits the aforementioned key advantages. This human-interface system has been designed to operate a Ground Control Station unit (GCS) for the remote control of Unmanned Aerial Vehicles (UAVs). For this kind of applications, the robustness of the system and the real-time performance are of paramount importance, guiding and restricting the system design.

## State of the art

Most of the works that perform real-time hand gesture recognition using the Leap Motion device use exclusively the provided hand-skeleton information, obtained by proprietary software. The common approach is to compute a feature descriptor from the hand-skeleton information, which is then processed by a classifier to recognize the final hand gesture. In [9], the trajectories of the hand-skeleton nodes are used to compute a descriptor, which is then delivered to a Hidden Conditional Neural Field (HCNF) classifier to recognize the gestures. In [10], a combination of the 3D Histograms of Oriented Optical Flow (HOOF) and Histogram of Oriented Trajectories (HOT) descriptors is used to characterize the hand gesture. Then, two different strategies are proposed to classify the computed descriptor into different gestures: a Support Vector Machine (SVM) and a Random Forest. In [11], trajectories from both hand skeletons are used to control dual robots. The obtained trajectories are filtered by an Interval Kalman Filter (IKF) and an Improved Particle Filter (IPF) leading to a better estimation of the hand position. In [12], a feature vector that encodes the skeleton information of fingertips, palm positions, and hand orientations is described. This feature vector is then delivered to a multiclass-SVM to distinguish among different hand gestures. In [13], distances and angles among fingertips and palm positions are used to compute a feature vector, which is delivered to an SVM classifier. In [14], an on-air text recognition application drawn by fingers is proposed, which combines Hidden Markov Models (HMM) and Bidirectional Long Short-Term Memory Neural Networks (BLSTM-NNs). In [15], a feature vector is computed using Euclidean distances and angles among fingertips. Also, pitch, roll, and yaw angles of the palm are encoded. For the classification stage: two different solutions are proposed, a multiclass SVM and a k-Nearest Neighbor (k-NN). In [16], a feature vector is computed using the Euclidean distance between consecutive fingers, and also among the fingertips positions. Then, an Artificial Neural Network (ANN) is used to distinguish among different hand gestures. Several Leap Motion devices are used in [17] to fuse their skeleton information via an HMM, and thus achieving a higher recognition performance in the American Sign Language.

Some works have also proposed to combine the skeleton information of the Leap Motion with imagery acquired by other devices, avoiding to use the Leap Motion imagery because of its strong geometrical distortion. In [18], a combination of depth imagery provided by the Kinect and skeleton information acquired by the Leap Motion are used to compute two different feature vectors. These are then delivered to a multi-class SVM classifier for the recognition task. In [19], color and depth information extracted from the Kinect device is combined with the skeleton information provided by the Leap Motion. A Convolutional Neural Network (CNN) is used as feature extractor for the depth and color imagery, while handcrafted features based on distances among fingertips are used for the skeleton information. Finally, an SVM classifier is used for the recognition task. Skeleton information from both the Kinect and the Leap Motion is acquired in [20]. First, one feature vector is computed from the skeleton of each device, encoding the information about the fingertip positions and their directions. Then, an HMM is used for the recognition process, considering different cases: feature vectors from each device are analyzed independently; features vectors are combined into one input for the HMM; lastly, a Coupled Hidden Markov Model (CHMM) manages the combination of the feature vectors. The Leap Motion has been also used in combination with non-visual devices. For example, the Leap Motion skeleton is fused with the information provided by the accelerometers of a wearable watch in [21]. A sensorized glove is also used in combination with the Leap Motion information in [22].

To the best of the authors’ knowledge, there is only one work that deals directly with the Leap Motion imagery [23]. This work starts by applying an image rectification process to correct the geometrical distortion of the images. In other words, to try to obtain a conventional-like image, such as those perceived by humans and acquired by most of the existing conventional cameras. Next, the hand regions are segmented and characterized by applying a Bag-of-Words strategy over Speeded Up Robust Features (SURF). Finally, a Radial Basis Function Neural Network (RBFNN) predicts the performed hand gesture. However, the result of the rectification process is far from being perfect (besides the additional computational cost), degrading the performance of the other stages. On the other hand, the system is not tolerant to failures: poor hand segmentations lead to erroneous recognition results. Lastly, the system is restricted to the recognition of static hand gestures.

In this paper, a novel real-time hand gesture recognition system based on the Leap Motion device is presented, which is able to recognize seamlessly static and dynamic gestures. This system processes the raw near-infrared imagery of the device, instead of the skeleton-based information, to achieve a higher accuracy and also to be independent of proprietary software solutions. The system is composed by three main modules: multi-proposal hand region segmentation, hand pose recognition, and hand gesture recognition. The multi-proposal hand region segmentation obtains a set of hand segmentation candidates per image, which are then characterized by feature vectors. Next, a structure of SVM classifiers predicts the most probable hand pose (a hand gesture is defined as a sequence of hand poses) in every time step by using the previous feature vectors. Lastly, a voting strategy is used to compute a feature vector that encodes a stream of predicted hand poses, which is delivered to a multi-class SVM to predict the final hand gesture.

Unlike the work in [23], that applies an image rectification process, an innovative strategy to deal with the geometrical distortion has been adopted. Since the main problem from a machine learning perspective is the higher intra-class variance (hand appearance changes according to the image location due to the distortion effect), a two-layer structure of classifiers has been proposed to alleviate it. The first layer builds a multi-class classifier structure by using a one-versus-all configuration of binary classifiers (everyone focused on one hand-pose class). The second layer implements every previous binary classifier as a bank of SVMs with the purpose that every SVM has to deal with subrange of visual appearances of a given hand pose.

Another novelty is the designed strategies to increase the robustness of the system at different levels. At the hand segmentation level, the robustness can be defined as obtaining a satisfactory enough hand segmentation that ultimately leads to a minimum recognition performance, independently of the performed hand gesture and the own acquisition process. Any kind of segmentation algorithm usually makes some assumptions (in this case regarding variables like light reflectivity, human skin intensity, or hand position with respect to the device) to obtain the correct segmentation. However, these assumptions are almost impossible to satisfy all the time in real conditions. This fact causes that the hand segmentation could be sometimes erroneous, and consequently the posterior hand pose recognition. For this reason, a changed of paradigm has been devised. Instead of obtaining only one segmentation that is assumed to be correct for the posterior hand pose recognition process, several hand segmentations are computed, assuming that at least one of them contains a hand segmentation with the enough quality to allow a successful hand pose recognition. The multiple hand segmentations are obtained by considering different situations and assumptions that can occur in real situations (some of them more probable than others). It is expected that several of these multiple segmentations are incorrect or not good enough, but these will be discarded in the recognition process, since their probability of being a specific hand pose will be very low. At hand gesture recognition level, the robustness can be defined as obtaining a successful hand gesture recognition from a sequence of recognized hand poses, even if a sequence has incorrectly classified hand poses. This can happen, for example, in extreme cases where the previous multi-segmentation strategy has failed in providing at least one correct hand segmentation, or because the multi-SVM classifier has not been enough trained. The proposed voting strategy precisely can detect and ignore those outliers (bad hand-pose detections) and predicts the more probable hand gesture recognition.

Another key contribution comparing [23], and most of the existing approaches, is that the system can naturally handle static and dynamic hand gestures.

Lastly, a new dataset has been created by including skeleton and near-infrared information that allows to evaluate the proposed system (that only uses near-infrared information) and be compared with other skeleton-based approaches of the state-of-the-art.

## Device characteristics

**Fig 1:**
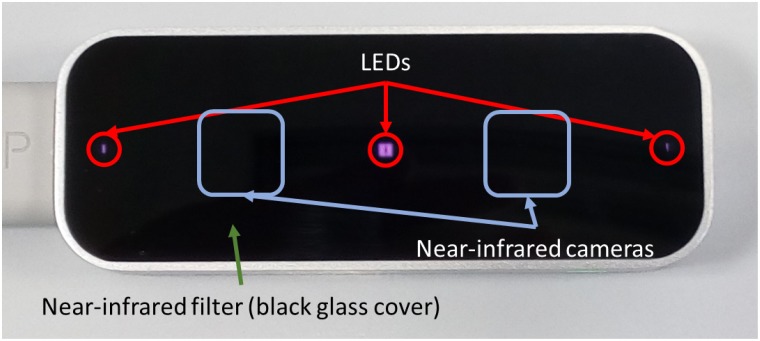
Leap Motion device showing where the LEDs and infrared (IR) cameras are placed.

The Leap Motion device is a consumer-grade device, especially designed for HMI applications. It has been conceived for close interaction using the hand motion in a contactless way. The device is able to estimate a hand skeleton for certain hand poses, and also to perform its tracking. The Leap Motion is an active device equipped with three LEDs that emit infrared light at a wavelength of 850 nanometers, and two near-infrared cameras sensible to the reflected infrared light (see Fig 1). Additionally, it has a near-infrared filter that makes that only the objects that are really close to the device (approximately, objects inside a volume of 1 m^3^ above the device) can be sensed (since they are well-illuminated by the LEDs). Thus, most of background objects are filtered by device design. Nonetheless, there can be clutter in the form of body parts: wrists, arms, heads, and chests, which are usually close to the hand.

The Leap Motion SDK provides the hand skeleton and the infrared imagery of the scene. The skeleton information consists on the position and velocity of the fingertips, the palm center, the wrist position, the finger lengths, and the location of the finger intermediate joints. Regarding the near infrared imagery, the Leap Motion provides two streams of images, one per camera, with a resolution of 640 × 240 pixels at a variable frame rate between 30 and 200 fps. The images are affected by a huge distortion due to the wide-angle lenses of the cameras.

### Hand reflectance characterization

**Fig 2:**
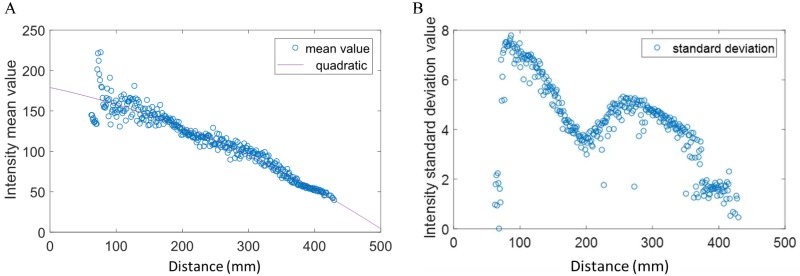
Mean and standard deviation intensity value of the hand regions with respect to the distance to the device. (A) Mean value, (B) standard deviation value.

The intensity values of the hand regions in the near-infrared images depend on the amount of infrared light reflected by the hands, which in turn depends on the distance between the hands and the device. In order to improve the hand location and its segmentation from the background, a study about the relationship between the intensity level of the hand regions and the hand-device distance has been conducted. For this purpose, the palm of the hand has been placed parallel to the upper face of the device at different distances, acquiring a set of images per every measured distance. Next, all the acquired images have been manually annotated indicating the hand location. This information has been used to compute the mean and variance of the intensity value of the hand regions as a function of the distance to the device. Fig 2 shows a graphic that plots the mean and variance of the intensity value according to the distance. As expected, the hand mean intensity value decreases with the distance following a quadratic behavior. As can be observed, the active area of the Leap Motion is between 100 mm and 440 mm. Below 100 mm, the device is blind by the hand reflection, and beyond 440 mm, the device cannot distinguish the hand from the background. The behavior of the standard deviation is more complex, revealing different working modes of the device. At the beginning, the value decreases with the distance, but then the value increases from 200 mm up to 280 mm, decreasing again from that distance on.

It is concluded from this reflectance study that the reflectance (mean value) of the hand decays quadratically with respect to the distance between the hand and the Leap Motion. On the other hand, the standard deviation of the hand reflectance does not decay in the same way, having a more complex behavior. The reason is probably the automatic gain control of the infrared LEDs that the Leap Motion has to try to preserve the signal-to-noise ratio.

The hand-reflectance profile (mean and standard deviation values) has been used to adaptively propose several segmentation candidates, as described in Section Multi-proposal hand segmentation.

## System description

**Fig 3:**
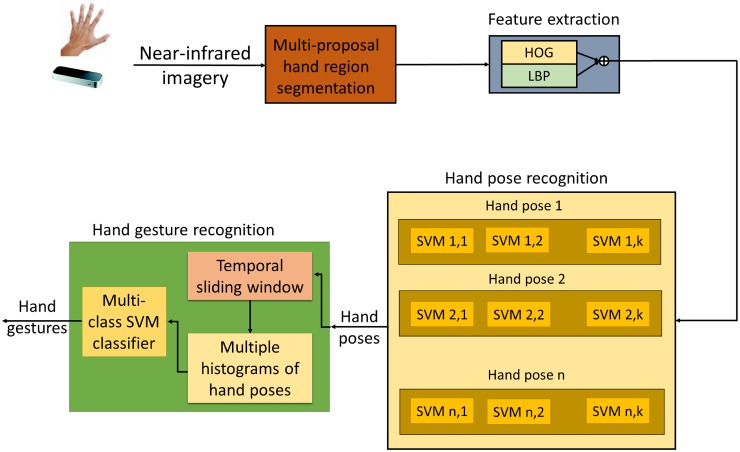
Block diagram of the proposed system.

Typically, a recognition solution is divided into a first step performing object segmentation, a second one performing the feature extraction process, and a third step in charge of the classification process. In the case of a dynamic object recognition algorithm, the third stage can additionally be split into two steps, one performing a recognition at each time step, and a second one addressing the temporal-based classification. Following this convention, the proposed hand gesture recognition system is divided into four stages (see Fig 3). The first one is a multi-proposal hand segmentation stage that computes several putative hand region segmentations. The second stage computes a feature vector from every candidate hand region, by combining Histogram of Oriented Gradients (HOG) and Local Binary Patterns (LBP) descriptors. The third stage predicts the hand pose using a two-layer structure of classifiers, which especially addresses the high intra-class variance produced by the strong geometrical distortion of the Leap Motion images. The fourth and last stage recognizes static and dynamic hand gestures from the previous stream of predicted hand poses. More specifically, a sliding window in the temporal domain selects a range of consecutive detected hand poses. Then, a set of histograms of hand poses is computed, which are finally stacked to create a hand-gesture descriptor. Lastly, a multi-class SVM classifier predicts the hand gesture from the previous descriptor of hand poses.

### Multi-proposal hand segmentation

The multi-proposal hand-segmentation stage computes several putative hand region segmentations with the purpose that at least one of them represents a satisfactory hand segmentation. This allows the system to be robust to deficient hand segmentations, which is a common problem for any visual gesture recognition system. This is accomplished by proposing a variation of the Otsu’s algorithm [24] along with the device characterization described in the Hand reflectance characterization Section.

**Fig 4:**
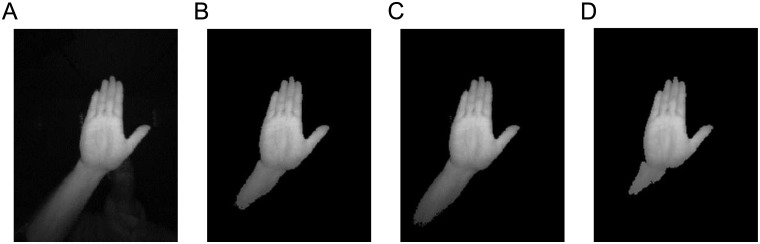
Hand image segmentation with three different thresholds. (A) original image, (B) result with Otsu’s threshold, (C) result with Otsu’s threshold plus one time the standard deviation, and (D) result with Otsu’s threshold minus one time the standard deviation.

The Otsu’s algorithm can generate satisfactory segmentations when the image can be clearly divided into foreground and background in some feature space. For this purpose, it estimates an optimum threshold that divides the feature space into those two clusters (foreground and the background) by minimizing the total intra-class variance of the clusters. However, the direct application of the Otsu’s method to segment the acquired images into hands and background using the intensity feature space would produce frequent errors, degrading the posterior hand gesture recognition. To solve this problem, multiple hand segmentations are computed by applying a probabilistic approach to the Otsu’s method with the purpose of computing at least one hand segmentation with enough quality. The multiple hand segmentation is modeled by a Gaussian distribution, where every hand segmentation candidate/sample is obtained by drawing a threshold value from this Gaussian distribution. The mean value is the original threshold obtained by the Otsu’s method, and the standard deviation is obtained from the hand reflectance characterization shown in Fig 2. An example of the segmentation stage is shown in Fig 4, where three thresholds are represented: one corresponding to the original Otsu’s algorithm, another adding one time the standard typical deviation, and another subtracting one time the standard typical deviation. Observe how the hand is segmented along with different forearm portions. The partially segmented forearm does not represent a problem for the recognition system, since the training images are subject to the same segmentation processing, and therefore the involved classifier learns that the hand gesture can include some forearm regions.

The hand reflectance characterization is also used to discard extreme abnormal segmentations by comparing the mean intensity value of a segmented hand region with the value resulting from the previous study. This mechanism, for example, can discard those abnormal segmentations resulting from situations where other objects than hands are present.

### Feature extraction

A feature vector is computed for every hand image segmentation by combining the Histogram of Oriented Gradients (HOG) [25] and the Local Binary Pattern (LBP) [26] feature descriptors. More in detail, the feature extraction stage can be divided into four steps: hand segmentation normalization, block division of the hand region, computation of feature descriptors per block, and computation of the final hand descriptor.

The first step, hand segmentation normalization, normalizes every hand segmented region to have a common size and aspect ratio in order to compute feature vectors of the same length, independently of the segmented region dimensions.

**Fig 5:**
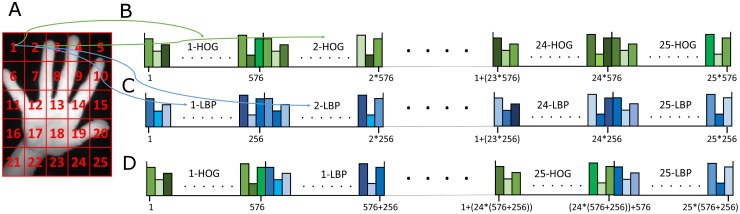
Block division and HOG and LBP feature vectors.

The second step, block division of the hand region, splits the normalized segmented region into *N*_*bl*_ = 5 × 5 = 25 non-overlapping blocks, as shown in Fig 5A. This block division will enrich the final hand region descriptor with valuable local spatial information. The number of blocks has been selected as a tradeoff between the length of the vector and its discriminative power: more blocks implies a higher discriminative capability, but also a higher dimensional feature vector.

The third step, computation of feature descriptors per block, characterizes the structural content of every block by means of the HOG and LBP descriptors. HOG describes a block by means of a set of local histograms that count the occurrences of gradient orientations, achieving a great robustness to illumination and contrast changes. The original HOG descriptor parameters have been used: input block divided into *N*_*sb*_ = 16 sub-blocks, sub-blocks divided into *N*_*c*_ = 4 cells, and *N*_*bi*_ = 9 bins per cell histogram. This configuration generates a final HOG histogram of dimension *N*_*HOG*_ = *N*_*sb*_ × *N*_*c*_ × *N*_*bi*_ = 16 × 4 × 9 = 576 components per block (Fig 5B). On the other hand, the LBP is based on the computation of histograms of intensity differences among pixels in a local neighborhood, and it is also very robust to dramatic illumination changes. The classical configuration of the LBP descriptor uses a neighborhood of 8 pixels and 1 bit to encode the neighborhood differences (the sign), resulting in histograms of *N*_*LBP*_ = 2^8^ = 256 components per block (Fig 5C).

The last step, computation of the final feature vectors, stacks the HOG and LBP feature vectors of all the blocks, obtaining a final descriptor of *N*_*bl*_ × (*N*_*HOG*_ + *N*_*LBP*_) = 25 × (576 + 256) = 20800 components, as shown Fig 5D.

### Classification of hand poses

**Fig 6:**
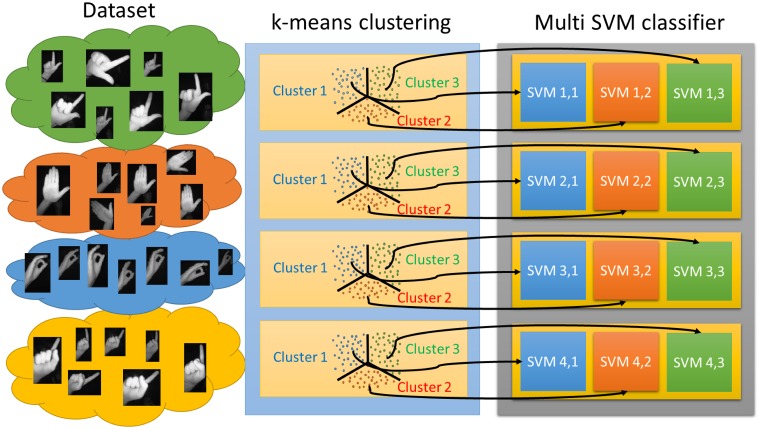
Example of the hand pose classification strategy using four hand poses and three different SVMs per pose, obtained by a k-means strategy.

For the hand-pose classification process, a novel two-layer structure of classifiers is used, which deals with the problems derived from the strong geometrical distortion of the Leap Motion images. One of the main effects of the distortion is the dramatic change in the aspect ratio of the bounding boxes that encloses a hand region, besides the own appearance itself. This fact makes the learning process of a classifier very complex, since very dissimilar feature descriptors can represent the same entity class. The proposed two-layer structure of classifiers precisely addresses this high intra-class variance. The first layer builds a multi-class classifier structure by using a one-versus-all configuration of binary classifiers (everyone focused on one hand-pose class). The second layer implements every previous binary classifier as a bank of binary SVMs that deals with the extra degree of freedom in the hand appearance due to the geometrical distortion. Thus, every SVM is in charge of a subrange of the whole range of bounding box aspect ratios associated to one specific hand pose. The number of SVMs is set to *K*, which are automatically configured by using a k-means clustering strategy, according to the aspect ratio, that partition the sample space of every hand-pose class into clusters of minimum variance (see Fig 6). Every SVM is trained using as positive samples those belonging to one cluster of one pose, and as negative samples the rest of the samples of the same pose and also all samples of the other poses (following the one-versus-all strategy). The number *K* of clusters has to be carefully chosen so that every cluster contains a sufficient number of samples to properly train all the classifiers.

As a result, an array of SVMs of size *K* × *N*_*c*_ is obtained, where *N*_*c*_ is the number of hand poses to be recognized. Thus, the output is a *K* × *N*_*c*_ binary array indicating the belonging to different possible ranges of hand appearances associated to every pose.

Lastly, to improve the recognition score, a non-linear Hellinger kernel [27], more commonly known as Bhattacharyya distance, is used for every SVM classifier.

The final output of the hand-pose classification stage is a set of arrays of hand-pose detections, one per hand-segmented region.

### Dynamic hand gesture recognition

The dynamic hand gesture recognition stage computes first a temporal feature vector of hand poses, and then uses an SVM classifier to finally recognize hand gestures, independently if they are static or dynamic.

**Fig 7:**
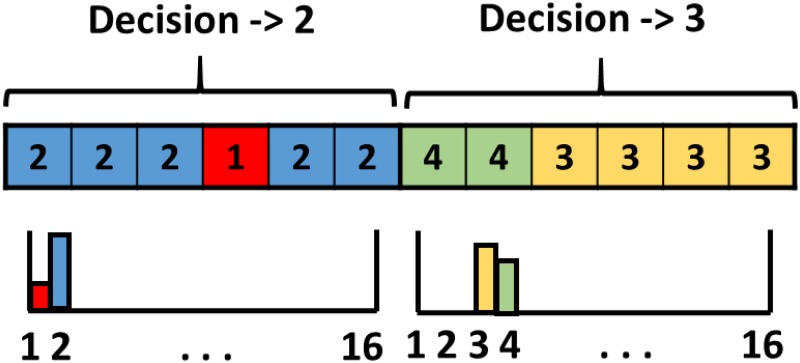
Example of the robust temporal feature vector of hand poses.

The temporal feature vector of hand poses considers a sliding window in the temporal domain to compute a moving histogram of hand poses. Thus, a histogram contains the distribution of multiple detected hand poses of the last frames (the length of the temporal sliding window). This histogram compactly represents both dynamic and static hand gestures. To increase the discriminative capability of the temporal histogram, multiple histograms can be considered inside the temporal sliding windows, which introduces more temporal information useful to encode the temporal order of consecutive hand poses. Fig 7 shows an example of the obtained hand-pose histogram considering two concatenated sub-histograms per temporal sliding window. As can be observed, most of the detections of the first half belong to the hand-pose number 2, while most of the detections of the second half correspond to the hand-pose number 3. Notice that the multi-histogram-based feature vector is robust to erroneously detected hand poses (different from 2 and 3 in Fig 7). These errors can be originated from either transitions between hand poses, or just errors in the hand pose recognition stage.

Finally, the previous multi-histogram-based feature vector is delivered to a multi-class SVM classifier to recognize the specific performed hand gesture. To avoid errors derived from situations without gestures or unknown ones, an additional class for that purpose is considered in the classifier.

## Dataset

To the best of the authors’ knowledge, there is not publicly available dataset with infrared imagery acquired by the Leap Motion device. The existing ones have only skeleton information. As a consequence, a new dataset has been created using the Leap Motion device to validate the proposed hand gesture recognition system, which includes both kinds of information, skeleton and infrared imagery. The dataset is publicly available in http://www.gti.ssr.upm.es/data/MultiModalHandGesture_dataset under the name of “Multi-modal Leap Motion dataset for Hand Gesture Recognition”.

**Fig 8:**
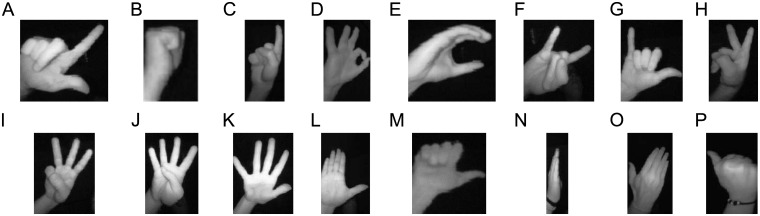
Hand poses of the dataset. (A) L, (B) fist moved, (C) index, (D) ok, (E) C, (F) heavy, (G) hang, (H) two, (I) three, (J) four, (K) five, (L) palm, (M) down, (N) palm moved, (O) palm up, (P) up.

**Fig 9:**
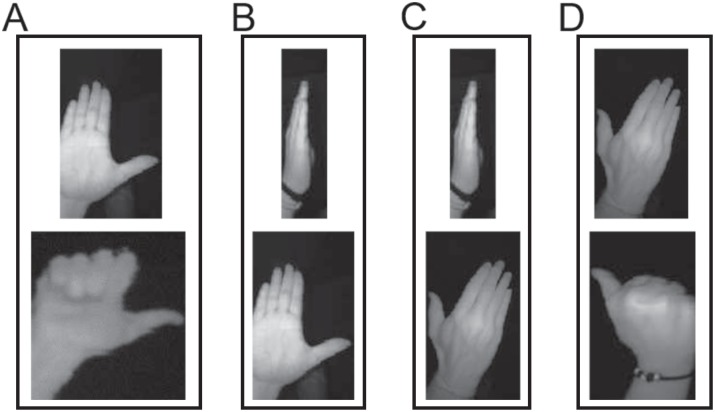
Dynamic hand gestures of the dataset. (A) go down, (B) go left, (C) go right, (D) go up.

For every temporal step, the Leap Motion device acquires two infrared images (one per camera) and the following skeleton information: hand and finger locations, data related with the finger extension, position of the wrist and palm center, and data about different finger parts. The dataset is composed of 16 different hand poses (see Fig 8) and 4 dynamic hand gestures (see Fig 9). A total of 25 different subjects (8 women and 17 men) performed each gesture of the dataset multiple times. All of them are laboratory employees with ages ranging from 22 up to 60 years old. All users have agreed to have their anonymized hand data and images shared in a public dataset. The static gestures have only one kind of hand pose, while the dynamic ones have two different kinds of hand poses. For the image acquisition process, each subject was sat in front of a computer with the Leap Motion placed horizontally on the table/console between the subject and the computer. Each subject was free to move the right hand over the device inside the Leap Motion field of view. A total number of 30 repetitions for every dynamic gesture were acquired by each subject. In the case of static gestures, a total number of 200 frames were obtained per gesture, and then divided into 20 different static gesture sequences. An image-mirrored version of the dataset can be used to train the system for left-handed people.

## Results

The proposed hand gesture recognition system has been evaluated using the hand gesture dataset introduced in Dataset Section, and compared with other state-of-the-art works [10] [13] [23] [18] previously introduced in the State of the Art Section, which make use of the Leap Motion device. Most of these algorithms only use skeleton information, excepting [23] that uses near-infrared imagery. The ultimate goal of the comparison is to evaluate the performance of every algorithm independently of the used data: only skeleton information or only imagery data. The dataset has been split into testing and training sets, where the 80% of the hand gesture sequences have been used for training, and the other 20% for testing.

The metric used for evaluating the recognition performance is the confusion matrix (CM), which represents the summary of prediction results on a classification problem, where each row represents the instances in an actual gesture class, and each column represents the instances in a predicted gesture class. From CM values, three different measurements have been collected: precision (p), recall (r) and F-score (F), whose mathematical expressions, expressed in percentage, are

p=100×tp/(tp+fp), (1)

r=100×tp/(tp+fn), (2)

F=2×p×r/(p+r), (3)

where tp represents true positive samples, fp the false positive samples, and fn the false negative samples. The F-score value is especially useful for comparison with other works, since it is a combination of the previous measurements that summarizes the algorithm performance.

**Fig 10:**
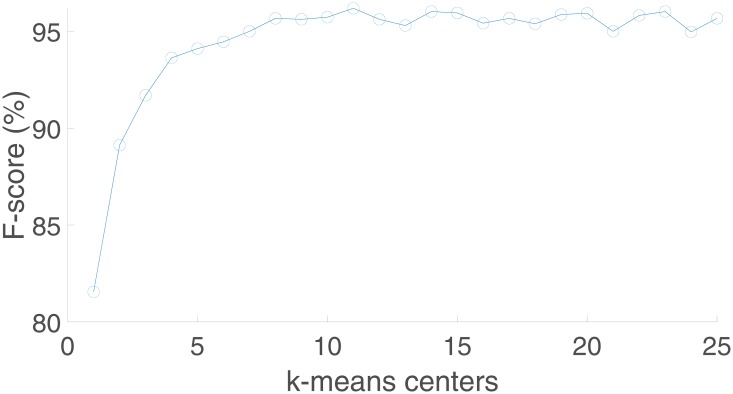
Evolution of F-score with respect to the number of k-means clusters for the hand pose recognition.

**Fig 11:**
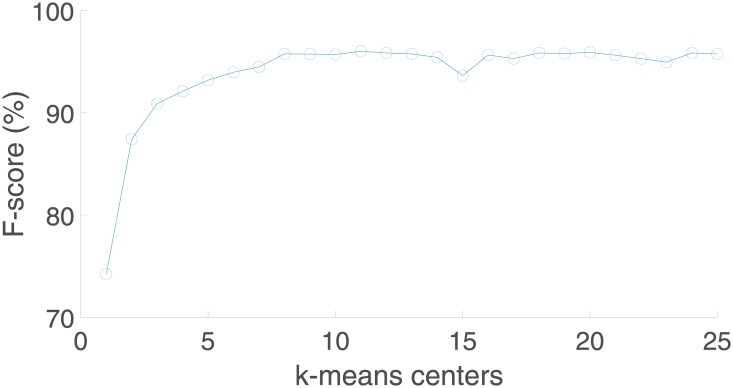
Evolution of F-score with respect to the number of k-means clusters for the hand gesture recognition.

Fig 10 shows the evolution of the F-score with respect to the number of k-means clusters for the hand pose recognition, reaching the maximum recognition performance for a number of clusters equal to 11. Observe that the k-means clustering strategy improves F-score from around 83% (without clustering strategy or one cluster) up to near 96%. On the other hand, the score achieved by using more than 11 clusters does not vary considerably, although increases significantly the performance time, especially in the training process. Similarly, Fig 11 represents the evolution of F-score for the hand gesture recognition using different number of k-means clusters. A similar improvement is obtained using the cluster strategy. Observe that the evolution of this curve is quite similar to the previous one, indicating the relevance and impact that a good pose recognition has over the whole hand gesture recognition system.

Table 1 contains the CM results of the recognition of hand poses for the proposed algorithm. As can be observed, the recall values, corresponding to the main diagonal, are greater than 92%, indicating that the system can predict correctly most of the time the different hand poses. Even more, the small variance among those recall values indicates that the system prediction is quite stable across the considered hand poses.

**Table 1:** Confusion matrix for the hand pose recognition, in percentage value.

	**palm**	**L**	**fist m**	**down**	**index**	**ok**	**palm m**	**C**	**heavy**	**hang**	**two**	**three**	**four**	**five**	**palm u**	**up**
**palm**	**95.13**	0.14	0.72	0.83	0.14	0.00	1.00	0.58	0.00	0.00	0.14	0.00	0.53	0.34	0.67	0.10
**L**	2.19	**95.55**	0.14	0.27	0.00	0.14	0.00	0.00	0.75	0.00	0.96	0.07	0.00	0.00	0.00	0.00
**fist m**	0.00	0.00	**97.29**	0.00	0.00	0.00	0.00	0.21	0.00	0.00	0.00	0.07	0.00	0.00	0.14	2.30
**down**	1.45	0.34	0.00	**95.09**	0.00	0.99	0.30	0.10	0.40	0.00	0.10	0.40	0.00	0.10	0.00	0.80
**index**	0.00	0.00	0.00	0.00	**96.22**	0.14	0.00	0.00	1.55	0.78	0.00	0.14	0.00	0.00	0.14	0.99
**ok**	0.26	0.56	0.26	0.06	0.45	**97.05**	0.00	0.00	0.00	0.00	0.32	0.00	0.00	0.58	0.32	0.06
**palm m**	1.73	0.00	0.77	0.05	0.34	0.34	**94.42**	0.24	0.43	0.05	0.00	0.00	0.05	0.00	1.59	0.00
**C**	0.00	0.00	0.51	0.22	0.07	0.58	0.00	**98.25**	0.00	0.22	0.00	0.00	0.00	0.00	0.00	0.15
**heavy**	0.15	0.37	0.00	0.00	1.19	0.00	0.07	0.07	**96.30**	0.00	0.00	0.96	0.30	0.00	0.59	0.00
**hang**	0.08	0.16	0.08	0.00	0.23	0.00	0.00	1.71	1.09	**95.47**	0.00	0.31	0.85	0.00	0.00	0.00
**two**	0.00	0.00	0.39	0.00	3.31	0.08	0.00	0.15	0.00	0.00	**95.90**	0.00	0.00	0.15	0.00	0.00
**three**	0.00	0.00	0.00	0.32	0.48	0.00	0.00	0.00	0.00	0.56	0.00	**98.34**	0.00	0.00	0.00	0.32
**four**	0.00	0.08	0.00	0.00	0.00	0.00	0.00	0.00	1.46	2.11	0.57	0.00	**95.75**	0.00	0.00	0.08
**five**	0.00	0.00	0.00	0.00	0.08	0.00	0.00	0.00	0.08	0.55	0.00	0.63	0.00	**98.55**	0.00	0.00
**palm u**	0.00	0.00	0.55	0.00	0.20	0.00	3.83	0.00	0.00	0.00	0.00	0.00	0.25	0.00	**92.95**	2.22
**up**	0.00	0.00	2.39	0.00	0.10	0.00	0.00	0.00	0.00	0.10	0.00	0.00	0.00	0.66	0.00	**96.75**

Table 2 shows the F-score results of the hand gesture recognition (static and dynamics) using different algorithms of the state-of-the-art, and also different variations of the proposed system. The comparison with the works [23], [10], [18], and [13] are performed considering only static hand gestures, since none of them is able to recognize dynamic hand gestures. This is indicated by a line in the involved cells of the table. As can be observed, the proposed system achieves the best results by a wide margin. Even the proposed variations that only used HOG o LBP (instead a combination of both) for describing every hand-segmented region achieved a much better score than the existing works in the state of the art. This fact proves the proposed contributions of using near-infrared images and the designed robust strategies of multi-hypothesis segmentation and voting-based recognition. It is also noticeable that the performance with some gestures using the skeleton-based solutions presented in [10] and [13] are very poor, below 20%. This is due to the Leap Motion not being able to compute a satisfactory skeleton for those gestures, a limitation not present in the works dealing with near-infrared images. In the case of the results obtained by [13], hand gestures for the “index” and “two” classes are badly recognized, because the Leap Motion is not able to correctly identify which finger or fingers are extended. Similarly for the results obtained by [10], the recognition performance of the “two” gesture is very poor, being confused with the “index” gesture. This behavior is different when using near-infrared images, as the results obtained by Runquing [23] illustrate. The recognition performance presented in that work is quite stable across classes, indicating that there are not limitations in recognizing specific hand gestures.

**Table 2:** Hand gesture recognition performance comparison with state-of-the-art works. In bold, the highest score per row.

	(Ours)HOG+LBP	(Ours)HOG	(Ours)LBP	RUNQING [23]	SCHMIDT [10]	MARIN [18]	CHUAN [13]
**L**	90.71	**99.93**	92.51	76.81	65.57	80.15	52.56
**fist m**	94.41	**99.78**	98.72	67.73	67.06	68.50	58.98
**index**	**98.57**	94.36	83.10	62.47	53.40	75.81	14.71
**ok**	**94.68**	90.31	71.48	51.41	50.02	40.77	31.34
**C**	96.21	**99.55**	96.10	61.73	44.10	34.44	78.74
**heavy**	**98.16**	94.35	75.91	72.97	41.19	59.45	60.31
**hang**	**96.86**	96.41	63.00	78.19	48.28	79.85	42.79
**two**	**95.71**	85.73	59.90	72.12	5.47	87.34	28.16
**three**	**97.48**	80.73	61.31	58.61	93.76	56.12	83.48
**four**	**95.24**	89.23	44.38	64.59	65.52	53.78	90.68
**five**	**96.19**	96.06	92.22	67.82	78.36	65.88	63.55
**go down**	**94.73**	93.39	89.10	—	—	—	—
**go left**	94.04	**96.97**	92.70	—	—	—	—
**go right**	**97.96**	93.61	91.54	—	—	—	—
**go up**	**99.33**	97.38	95.48	—	—	—	—
**Mean**	**96.02**	93.85	80.50	66.77	55.70	63.83	55.03

The computational cost of the proposed system has been measured to prove its real-time operation using a laptop computer with an i7-3540M processor. Notice that no specialized hardware (such as a Graphical Processor Unit, Digital Signal Processor, or Field Programmable Array) has been used. Neither a multi-core / multi-threading implementation has been developed. Regarding the concept of real-time operation applied to the context of human-computer interaction, it can be defined as the capability of recognizing hand gestures with a minimum latency / delay that allows the user to naturally interact with a machine without uncomfortable or unproductive waiting times. For this purpose, the entire recognition process (from segmentation up to gesture classification) should take less than 100 milliseconds (ms) [28]. Table 3 shows total and partial computational times, where time measurements have been averaged over 200 repetitions for different dynamic hand gestures. The proposed system can process a whole sequence of images with a latency of 31.3 ms, achieving widely the goal of real-time performance. Regarding the computational times per stage, the segmentation process takes 8.15 ms, the computation of HOG and LBP feature vectors 6.59 ms, the hand pose classification 16.51 ms, and the hand gesture recognition 0.05 ms.

**Table 3:** Time performance.

Process	Time (ms)
**Segmentation**	8.15
**Features computation**	6.59
**Pose classification**	16.51
**Gesture recognition**	0.05
**Total system**	31.3

## Conclusions

A real-time hand gesture recognition system based on the use of near-infrared imagery acquired by the Leap Motion device has been presented. Unlike other works, no skeleton information is used for the recognition process, only infrared imagery. This fact avoids to use proprietary software and also increases the hand gesture recognition performance, since it avoids problems related to the hand skeleton estimation. The system starts by computing hand segmentation proposals, which are then characterized by a combination of feature descriptors (HOG and LBP). These are delivered to a two-layer structure of classifiers, which especially addresses the high intra-class variance produced by the strong geometrical distortion of the Leap Motion images. As a result, the recognition of individual hand poses is achieved, which are then used to build a temporal histogram of hand poses. Finally, another multi-class SVM recognizes the performed hand gesture using the previous histograms of hand poses. The achieved recognition scores indicate the advantages of using near-infrared imagery instead of using skeleton one, both acquired by the Leap Motion device.
